# The BRIDGE project: a feasibility randomised controlled trial of brief, intensive assessment and integrated formulation for young people (age 14-24) with features of borderline personality disorder (Protocol)

**DOI:** 10.3389/fpsyt.2024.1389578

**Published:** 2024-09-24

**Authors:** Ruchika Gajwani, Fiona Sim, Kirsty McAllister, Helen Smith, Emma McIntosh, Paul Moran, Dennis Ougrin, Michael Smith, Andrew Ian Gumley, Andrew M. Chanen, Helen Minnis

**Affiliations:** ^1^ School of Health and Wellbeing, University of Glasgow, Glasgow, United Kingdom; ^2^ National Health Service Greater Glasgow and Clyde, Glasgow, United Kingdom; ^3^ National Health Service (NHS) A&A, Ayrshire & Arran, Scotland, United Kingdom; ^4^ Department of Population Health Sciences, University of Bristol, Bristol, United Kingdom; ^5^ Youth Resilience Unit, Wolfson Institute of Population Health, Queen Mary University of London, London, United Kingdom; ^6^ Orygen, Melbourne, VIC, Australia; ^7^ Centre for Youth Mental Health, The University of Melbourne, Melbourne, VIC, Australia

**Keywords:** borderline personality disorder (BPD), BPD features, early intervention, young people, feasibility RCT (fRCT), community intervention, Cognitive Analytic Therapy (CAT)

## Abstract

**Background:**

Borderline personality disorder (BPD) is a severe mental disorder that is characterised by a pervasive pattern of instability of interpersonal relationships, self-image, and mood as well as marked impulsivity. BPD has its peak incidence and prevalence from puberty through to emerging adulthood. BPD is a controversial diagnosis in young people. Commonly, young people with BPD are under-diagnosed, untreated, are not in employment or training and are estranged from their families. Yet, they have complex needs and are at high suicide risk.

**Aim:**

To assess the feasibility of conducting a randomised controlled trial (f-RCT) of a BRIDGE, a brief intervention programme for young people (age 14-24) with BPD symptoms (sub-threshold or threshold) in a community sample from Scotland, UK.

**Method:**

Forty young people (age 14-24) meeting criteria for BPD symptoms, will be randomised in a 1:1 ratio to receive either a) the BRIDGE intervention plus service-as-usual or b) service-as-usual alone. Follow up will be 12 weeks and 24 weeks post-intervention. The study is carried out between 2021 to 2024.

**Outcome:**

The two primary outcomes of the MQ funded, BRIDGE project (f-RCT) are i. recruitment rates and ii. retention rates. The study will present the acceptability and appropriateness of our putative outcome measures for a future definitive randomised controlled trial (d-RCT).

**Impact:**

Young people with BPD benefit from good clinical care and targeted intervention, however are regularly missed or mislabelled. The community based feasibility trial would provide initial evidence of variable needs of young people with complex needs, who maybe missed or excluded from services as they don’t “fit” a model/diagnosis. Workable multi-agency service model proposed in the trial would be a major advance in understanding care pathways regardless of trial outcome.

**Clinical Trial Registration:**

ClinicalTrial.gov, identifier NCT05023447

## Introduction

Borderline personality disorder (BPD) is a severe mental disorder that is characterised by a pervasive pattern of instability of interpersonal relationships, self-image, and mood as well as marked impulsivity. BPD has its peak incidence and prevalence from puberty through to emerging adulthood (1, 2). The adverse personal, social and economic consequences of BPD during this developmental period have been summarised in many publications (3, 4). In brief, BPD among young people uniquely predicts severe and harmful current problems, increases the complexity of clinical presentation and treatment delivery and acts as a gateway to diverse and serious problems later in life that includes a mortality rate 10 times that of general population and an almost two-decade reduction in life expectancy (5–7). Moreover, those who care for and about these young people also experience severe difficulties (8).

Despite these compelling facts, along with good evidence that early intervention can improve these short- and long-term outcomes (9), BPD is a controversial diagnosis worldwide when applied to young people (10–12). This is largely because the condition is associated with a high degree of public, self, structural and institutional stigma (13, 14). This has commonly led to clinical cultures in which the diagnosis is avoided or delayed, or substitute diagnoses are used, in the belief that this is in the best interests of young people. In turn, this leads to delays in effective treatment, risks the use of inappropriate and/or ineffective treatments leading to poor outcomes and risks further stigmatising people living with the disorder (15).

Young people with the persistent and complex needs that are characteristic of BPD – i.e. those with the poorest functioning (4) and most complex psychiatric presentations - often find themselves falling out of contact with services by age eighteen (16). Barriers to diagnosis and treatment of BPD in young people include the widely held beliefs that a BPD diagnosis implies permanent disability, will lead to clinical and self-experiential discrimination (17), and to widening social, health and occupational disadvantage. Despite randomised controlled trial (RCT) evidence for the effectiveness of specialised psychosocial treatments for BPD in young people (9), the effect sizes for these treatments are modest and not uniformly sustained at follow-up. Furthermore, these specialised treatments are often complex and unlikely to be scalable at a population level within existing service provisions. Scalable approaches to mitigate against the short- and long-term adverse outcomes for young people living with BPD, for the benefit of the whole population, is a public health priority (18). Greater social and health inequality is bad for everyone (19). We have proposed elsewhere that early intervention programmes for severe mental illness may benefit from a developmental clinical staging model by assessing children and young people in greatest need of mental health services for more equitable service delivery (20).

Young people with early BPD benefit from good clinical care and targeted intervention (21), however their needs are often under-recognised. NICE guidelines for BPD recommend (22) that people with BPD should not be excluded from any social and health care services but, in practice, BPD sufferers experience of discrimination and disengagement from specialist services which perpetuates social and health inequalities (6, 23), and iatrogenic harm (24).

In this paper, we will outline the protocol of a feasibility randomised controlled trial (f-RCT) of a novel community based therapeutic intervention of brief, intensive assessment and integrated formulation (BRIDGE intervention). BRIDGE intervention development has been guided by a) the emerging evidence base for Cognitive Analytic Therapy with young people (age 15-25) with BPD in an early intervention service (25) and b) in collaboration with an established Glasgow intervention programme, called Intensive Support and Monitoring Service (ISMS; https://dera.ioe.ac.uk/id/eprint/9517/1/0064165.pdf), used in the Glasgow youth justice system. ISMS has the explicit focus of reaching a shared formulation with the young person and the multi-agency system that supports them.

The BRIDGE intervention delivers on three core elements: a) shared formulation of comprehensive psychiatric, health, functioning assessments, b) up to sixteen sessions of Cognitive Analytic Therapy, c) contextual work with the young person and multi-agency teams involved with the participant. With this in mind, this trial aims to establish the feasibility of engaging and retaining of young people with features of borderline personality disorder in the community for participation in the f-RCT. We also aim to map the complex and varied needs of this group of young people with BPD features, who may otherwise be missed from services because they don’t “fit” a traditional service model/diagnosis (26), and to examine the acceptability of the trial processes.

Our approach to understanding the complex social/service delivery for this group is informed by syndemics, the term given to the co-occurrence of multiple, inter-related health problems at the individual- and population-level, developing and being sustained by harmful/unhelpful social contexts. This approach (25) is our key methodology for examining and optimising interdisciplinary working. Syndemics is embedded within the research methodology, the multi-agency intervention approach, our international network of collaborators, and the central role of young people at all stages of study design, delivery and dissemination.

Societal and professional attitudes to personality disorders, and the service provision that follows from these attitudes, have an important part to play in *how* services are delivered. An important approach to furthering understanding of complex social/service delivery is through consultation with individuals with lived experience of borderline personality disorder has been a cornerstone of the development and ongoing refinement of this clinical trial. A Youth Advisory Group (YAG) comprising individuals from the Transdisciplinary Research for the Improvement of Youth Mental Public Health network (TRIUMPH http://triumph.sphsu.gla.ac.uk/) was established at the funding application stage of the trial and has offered consultation throughout the trial. Co-investigators with lived experience of BPD have provided insight and guidance to the acceptability of procedures (i.e. design and planned recruitment) on an ongoing basis.

## Methods

### Trial objectives

To assess the feasibility of conducting a randomised controlled trial (f-RCT) of BRIDGE, a brief intervention programme for young people (age 14-24) with BPD features (sub- or full-threshold), in a community sample of Glasgow, Scotland.

### Research questions

1. Are recruitment and retention rates adequate to suggest a future full-scale RCT would be possible (quantitative)?
2. What are the characteristics (in terms of mental health and service use) of the young people referred to the study (whether or not eventually randomised).
3. How acceptable are the trial processes and interventions to the participants (qualitative and Patient and Public involvement; PPI)?
4. Is it feasible to collect clinical and health economic outcome data for a future definitive randomised controlled trial (d-RCT) (missingness and data quality)?
5. Does the trial need adapting for participants in different settings (qualitative and PPI)?
6. Do trial processes need adapting in order to successfully recruit participants from different referral routes (quantitative and qualitative)?

### Trial design/settings

Our study design is a single-blind, parallel groups f-RCT following the Medical Research Council Complex Interventions Framework (27) over a period of 36 months. After a two-stage screening process (details below), eligible participants meeting diagnostic criteria for BPD features are invited to baseline research assessment, then randomised to BRIDGE and service as usual (SAU) or SAU (alone).Aspects of feasibility are explored using qualitative and quantitative methods.

The study is set in the city of Glasgow, Scotland’s largest and most ethnically diverse city with an estimated population of 635,130, of which almost 12% represent an ethnic minority. Adolescents and young people represent approximately 12% of the Glasgow population; over half live in areas in the two most deprived deciles within Scotland, while only 4% Glaswegian children live in areas in the least deprived decile within Scotland (https://www.gov.scot/collections/scottish-index-ofmultiple-deprivation-2020/).

#### Inclusion criteria

- 3 or more of the 9 DSM-5 BPD criteria (28) assessed using the SCID-PD. While clinical symptom remission has been a critical treatment goal of RCT’s, there is now compelling evidence for addressing the functional outcomes characteristic of BPD features at both subthreshold and threshold levels (9).
- Age 14-24 (inclusive).

#### Exclusion criteria

- Currently receiving evidence-based psychosocial treatment for BPD, with a minimum of six sessions attended prior to any disengagement/drop-out.
- Has received any previous evidence-based psychosocial treatment for BPD.
- Receiving specialist intensive psychiatric treatment at the time of study entry, for severe mental health disorder (such as DSM-V psychosis or severe anorexia nervosa).
- Insufficient fluency in English to complete the study protocol.
- Living out-with Glasgow City health board.

### Participant recruitment

Recruitment is taking place over 24-months, from October 2021 to September 2023. As per recommended Medical Research Council guidelines for f-RCTs (27), a formal sample size calculation is not appropriate. Our pilot work (29) suggests that we will achieve a sample size of forty adolescents and young people (age 14-24), with subthreshold or full-threshold BPD features, recruited from the community and randomised to each of the two arms of the trial. This sample will allow us to estimate the sample size for a phase III trial and the human resources required to achieve recruitment and retention.

We will also explore the feasibility of different routes of referral to the trial. Participants might or might not be involved with a mental health service provider to meet criteria for trial. However, there will be an expectation that all participants will be registered with a general practitioner (GP).Where a participant has no fixed abode, a GP will be identified within the homelessness GP team. Participants will be recruited through ethically approved channels that might include:

- Self-referral through advertisements in public places (i.e., transport, libraries, social media).
- Referral from professional within the NHS GG&C (specialist children’s services, Adult mental health services, GP, A&E).
- Referral from professional within social work, forensic services, youth support services.
- Referral from third sector organisations.

Professionals within each of the above services will be made aware of the study through presentations with the trial team. They will then be regularly asked if any patients on their caseload/service users meet inclusion criteria for the study and can be approached for participation. With potential participants, verbal consent will be sought and noted before referring for further participation with the clinical trial.

Symptomatic remission, occurring over a number of years, is a common feature of BPD (30). Yet, there is robust evidence to support that young people who meet subthreshold criteria for persistent, long-standing difficulties characteristic of BPD features, also have psychiatric comorbidity and functional impairment and are likely to be referred to mental health services for disruptive or suicidal behaviour (31). To address the heterogeneity inherent in psychopathology (32) and within subsyndromal BPD (33), screening for randomisation of participants to BRIDGE intervention (plus SAU) or SAU would include a) enhanced phenotyping at phase 2 (below) which will ensure adequately defining the screened population and b) a robust measure of social function (i.e. KIDSCREEN-10) (34).

#### The screening will include a two phased approach

Phase 1 (screening): Participants (accessed through a range of settings in Glasgow) will self-complete the brief (15- item) SCID-II PQ-BPD questionnaire. Potential participants will be either referred by a professional in a range of settings in Glasgow or can self-refer: everyone will complete either an online (www.bridgeproject.co.uk) or paper screening assessment. Those meeting the cut off for the SCID-II PQ BPD (ref) (>11 out of 15) will be invited to Phase 2. SCID-II PQ-BPD has good psychometric properties and is used as a screening tool for BPD in outpatient youth (35).

Phase 2 (diagnostic screening): All potential participants will be invited for an interview conducted using the Structured Clinical Interview for DSM‐V Axis II Personality Disorders (SCID‐5-PD) BPD module. This will be carried out by a clinician or clinically trained researcher. Those young people who meet 3 or more SCID-5-BPD criteria (subthreshold or full-threshold) will be eligible for randomisation to the BRIDGE study.

### Intervention

Brief, intensive assessment and integrated formulation (BRIDGE) intervention development has been guided by the emerging evidence base for community based comprehensive assessment and brief intervention with young people with longstanding unmet needs characteristic of BPD features.

#### BRIDGE will be delivered over 3-6 months and has three foci

1. Comprehensive assessment, taking up to two sessions, including BPD symptoms, co-presenting difficulties, neurodevelopmental profile, life events history and psychosocial functional impact.
2. Up to 16 sessions of Cognitive Analytic Therapy (CAT) (36).
3. Develop a shared formulation with the young person, using CAT principles (Reformulation, Recognition and Revision) and their comprehensive assessment, and, where clinically applicable, their family and service-providers. Developing a shared formulation with the young person and multi-agency group would be unique to the participant, using contextual CAT.

### Treatment fidelity

A fidelity evaluation framework will be optimised to the specific needs of this complex intervention (37) with the focus on a) participant responsiveness as engagement and retention is the primary focus of the trial b) complex intervention adherence (Frequency, duration and nature of engagement) c) quality of delivery (protocol adherence, monitoring assessments and treatment delivery) d) identifying barriers and facilitators (recruitment, engagement, retention) e) outcome measurement of change and sustainability. Trial therapists will have two-weekly supervision with a senior CAT practitioner and supervisor, accredited by the Association of Cognitive Analytic Therapists (ACAT), with extensive experience of working with young people with BPD.

### Comparison (SAU)

For participants randomised to SAU, a routine letter acknowledging their participation will be shared with their service provider(s), including the GP. SAU, which is likely to range from social services, mental-health services, forensic services, to no intervention, will be documented for each participant. The intervention will be described in detail through the qualitative process evaluation.

There will be no formal measure of treatment fidelity for SAU.

### Procedure

Our three-month pre-trial set-up phase will establish a detailed system for managing referrals, randomisation, and networking with likely referral sources, including any self-referrals detailed in

**Figure 1:**
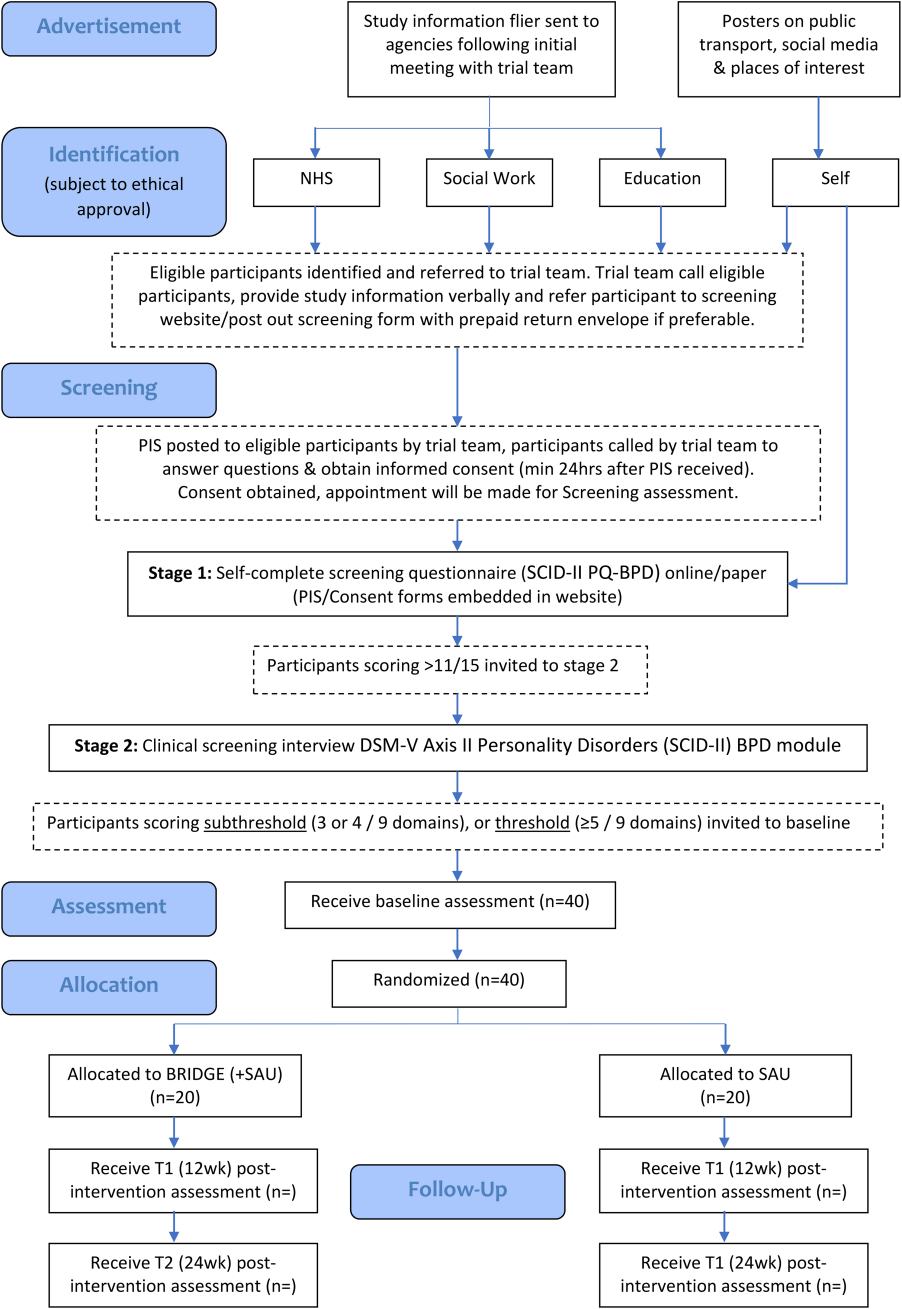
Participant flow diagram.

**Figure 1**. All potential participants will receive a ‘Participant Information Leaflet’ and a ‘Trial Consent Form’ prior to Screening 1. The trial research team will then invite young people meeting criteria at screening 1 for an assessment (Phase 2) to determine eligibility for subthreshold or full-threshold BPD features. The assessment will be conducted by a clinically trained researcher, prior to study randomisation.

Young people (age 14-24) or parents (for those aged 14-16 who are judged by a healthcare professional, i.e. GP, as incapable of providing written, informed consent) with child assent will complete the Participant Information sheet (online or paper format) and consent form. Capacity to consent will be confirmed and documented by a healthcare professional at diagnostic screening 2, prior to randomisation.

### Randomisation

Randomisation will be managed by the Trial Team at the University of Glasgow. Participants will be randomised (1:1) to either BRIDGE (+SAU) or SAU. A statistician from the Robertson Centre for Biostatistics at the University of Glasgow will generate a randomisation table. Research outcome assessors will be blind to treatment allocation. The randomisation list, containing the unique study ID and participant name, will be stored on a password protected file within the Trial Team’s private.

Microsoft Teams channel (following sponsor representative GDPR guidelines, at the university of Glasgow secure server). Access will be restricted only to those members of the research team who will remain unblinded throughout the study. A log of access will be maintained. Participants will be randomised by a member of the administrative team independent of the trial, using the randomisation procedure provided by the study statistician.

All participants consenting to participate will be invited to complete assessments, post-randomisation, at baseline, 12- and 24- weeks post-randomisation. A minimum data set of care pathways will be described at baseline. Follow-up of all participants will occur for the purpose of future modelling of health economics impacts in relation to quality of life and functional outcomes.

Case-study methodology will be used to understand individual contexts, involving embedded qualitative evaluation with about 10- 15 young people (five to seven from BRIDGE intervention and five to seven from SAU’s) and, where possible, their parents and relevant service providers. Topics will include acceptability of the three elements of BRIDGE, perceived mechanisms of change and exploration of requirements for data collection for a future d-RCT, including for future health economic evaluation.

### Measures

The only quantitative outcome measures in the f-RCT are recruitment (i.e. proportion of young people referred as eligible who are consented and randomised) and retention (i.e. proportion of those randomised who complete follow-up assessments at six months post randomisation).

We have examined the literature and best practice guidelines to select likely outcome measures for use in a future definitive RCT (d-RCT) (e.g., the International Committee for Harmonization of Outcome Measures for Personality Disorder (38). The f-RCT will investigate the acceptability and appropriateness of these measures for a future d-RCT which include, at screening 1/pre-randomisation: The Structured Clinical Interview for DSM‐V Axis II for Personality disorders (SCID-II PQ-BPD) consists of 15 questions which explore the presence of BPD symptoms using ‘Yes’ or ‘No’ answers. The SCID-II PQ-BPD has been found to have fair-to-good agreement with a SCID-II interview BPD diagnosis and good test–retest reliability in young people (35).

Should an individual score a minimum of 11 of the 15 questions they are invited to the Structured Clinical Interview for DSM‐V Axis II Personality Disorders (SCID‐5-PD) BPD module. This is a semi-structured interviewed which explores these 15 questions in depth measuring on a scale of?-3 to what extent an individual’s symptoms impact on their functioning. (? = inadequate information, 1= absent or false, 2 = subthreshold, 3 = threshold or true. A pervasive pattern of instability in relationships, self-image, mood, impulsivity, anger and the occurrence of dissociation are explored. Although the DSM-5 (39) used the criteria that five of these categories are met, for the purposes of BRIDGE, the presence of three or more BPD traits will be used as diagnostic inclusion criteria, as identifying early signs and symptoms is the objective.

In this second screening further measures are carried out. KIDSCREEN 10 (34) is a health questionnaire for children and young people, exploring how they have perceived their health to be in the previous week. The KIDSCREEN 10 (34) is a standardised assessment tool for health and its impact on quality of life, demonstrating good internal consistency (Cronbach’s alpha =.82), and good test-retest reliability (r=.73; ICC=.72) (40).

Participants will complete the following measures pre-randomisation: Pathways to care and demographic questionnaire, Mini International Neuropsychiatric Interview (MINI) 7.0.2 (18yrs+) and the Mini International Neuropsychiatric Interview (MINI-KID) 7.0.2 (under 18yrs) (41); The MINI is a diagnostic, structured tool for assessing common psychiatric disorders. Studies (42) have shown that the MINI has psychometric reliability and validity and can be delivered with short training input and in a brief period of time. Adverse Childhood Experiences Scale (ACES) (43) asks 10 questions regarding traumatic experiences that occurred before the age of 18. Autism Symptoms adolescents (ASSERT) (44)is a self-report tool with 8-questions pertaining to poor social understanding, rigidity and repetitive behaviours and interests. Adult ADHD Self-Report Scale (ASRS) (45) is an 18-question screening tool used by BRIDGE to screen for attention deficit disorder symptoms such as feelings, conduct and level of impairment in the previous 6 months. It has 2 subscales; 1) Inattention and 2) Hyperactivity. It has been found to be a reliable and valid scale, showing high internal consistency (Cronbach’s alpha 0.88) and rater-administration (Cronbach’s alpha 0.89) (46).

At baseline, 12- and 24- week follow-up, assessments include: Borderline symptoms list (BSL-23)

(ref) This 23-item list explores the impact of BPD symptoms in the course of the previous week (47).

The questions explored include concentration, emotions, vulnerability and overall daily functioning. A BSL supplement is also used in BRIDGE which assesses behaviours over the course of the previous week. The BSL-23 has excellent psychometric properties, high internal consistency (Cronbach = 0.97) and test-retest reliability (0.82) (48). Sheehan’s Disability Scale (SDS) (49) is a self-reporting tool which assess the impact of symptoms on functional impairment. There are three scales, 1) Work*/school, 2) Social life, 3) Family and home responsibilities. Days lost and days unproductive due to symptoms are also rated. Barratt Impulsiveness Scale (BIS-11) (50) is consists of 30 questions which consider 1) Attentional Facet, 2) Motor Facet, 3) Planning Facet. The Cronbach alphas range from 0.73 to 0.83 indicating good reliability. Difficulties in Emotional Regulation Scale-SF (DERS-SF) (51) consists of 18 questions and measures how an individual relates to their emotions such as understanding, awareness and acceptance. In terms of validity and reliability the DERS-SF had a Cronbach-Alpha range of 0.90-0.98 and 81-96% shared variance. Patient Health Questionnaire-9 (PHQ – 9) (52) is a self-report tool of 9-questions which monitors the severity of depression. It measures areas such as sleep, low mood and appetite. Quality of Life Questionnaire (EQ-5D 5L) (53) is a short self-report tool which asks participants to measure their health on that day on a scale of 5 levels - “no problems, I slight problems, moderate problems, severe problems and unable to carry out activity”.

Quality of Life will be assessed with ICECAP-A (54), with a Cronbach’s Alpha of 0.86. Suicidal

Ideation Scale (SIS) (55) is a 10-question 1-5 scale (1 being “never or none of the time and 5 being “always or a great many times”) which can detect early suicidal ideation among adolescent and young people in the general population. It was found to have excellent internal consistency and test-retest reliability.

The feasibility and acceptability of this battery will be explicitly assessed as one of the trial’s key feasibility parameters: qualitatively, we will examine the acceptability and appropriateness of these outcome measures in terms of whether or not participants think we are asking the right questions and whether the measures are acceptable or too burdensome. Prior to final decisions about use of measures in a future d-RCT, the measurement battery will also be discussed in the expert Scientific Advisory Group and Youth Advisory Group.

All the measures will be incorporated into a user-friendly questionnaire “book” and participants will have the option of completing on paper, in a telephone interview, or a mixture – a technique that has worked well in previous studies. These measures will be conducted by trained researcher assessors, blind to group status, at 12- and 24-weeks post-randomisation.

### Statistical analysis

Statistical analyses will be conducted by a statistician/researchers who are blinded to treatment allocation. Recruitment, retention rates and the BRIDGE sessions attended will be calculated with 95% confidence intervals (CI). A simple descriptive analysis of recruitment/retention relative to eligible/approached population will be conducted. While underpowered, between-group change in each measure after adjustment for baseline will be estimated using analysis of covariance (ANCOVA). The emphasis will not be on reporting of significance but variance in outcome measures between groups by reporting mean differences based on change from baseline with 95% CI and effect sizes reported. The standard deviation of the assessments for the putative outcome measures at baseline and follow-up will be estimated, shaping the future d-RCT. Statistical analysis plan and data sharing agreement will be agreed by the Scientific Advisory Group in consultation with the NHS Scotland’s Mental Health Network PPI group.

### Process evaluation

Interpretative Phenomenological Analysis will be used to explore participant’s experience of engagement and retention with the BRIDGE project. Process evaluation of the contexts and experiences of the young person presenting to the BRIDGE project will enable us to develop a framework for context‐mechanism‐outcome configurations (CMOCs) (56) that will be unique to the young person’s experiences and the multi-agency engagement (or the lack of) around them (57). This would involve qualitative interviews with ten to fifteen young people and, where possible, their parents and relevant service providers. Topics will include acceptability of the three elements of BRIDGE intervention, perceived mechanisms of change and exploration of requirements for data collection for a future d-RCT, including for future health economic evaluation.

### Health economics

For the economic analysis, data will be collected on cost of delivering the intervention, in addition to participant’s use of health, personal, and social services and broader educational and societal resources in the six months before study baseline data collection. Adolescent and young adult’s quality of life will also be measured within trial using the ICECAP-A (54). Measurement of quality of life is an important input for the economic component of this study and will enable assessment of any short-term change in quality of life for young people between baseline and 6 months, and also between the trial arms. The clinical and service use characteristics of young people referred to the study will be used to build a health economic logic model for potential lifetime impacts of BRIDGE.

### Evidence of feasibility: pilot study and expected results

This proposal builds on our previous *Pathways study* (29), (2016 – 2018; funded by NHS GGC): majority of recruits came from Child and Adolescent Mental Health Services (CAMHS); recruitment barriers included client disengagement with specialist services before meeting the research team, or clinicians considering patients as high risk to take part. Yet, once consented to the study, there were no withdrawals and the completion rate of the assessments was high, with incomplete data on only 4% of participants. Our work with *Pathways* has shown that multi-modal recruitment methods, with excellent service user consultation, can achieve high recruitment rates of young people considered hard-to-reach. In the BRIDGE project, we expect similar barriers to engagement/referral from services. We expect data from eligible participants to demonstrate functional difficulties across different facets of life in those presenting with subthreshold and threshold BPD. The development of workable multi-agency services proposed in the trial would be a major advance in optimising care pathways, regardless of trial outcome.

## Discussion

Young people with BPD benefit from evidence-based psychosocial interventions. However, they are infrequently identified or are excluded from services. NICE guidelines for BPD recommend that people with BPD should not be excluded from any social and health care services. Yet, in practice, discrimination and disengagement from specialist services perpetuates pre-existing social and health inequalities. We aim to identify the feasibility of recruiting and engaging adolescents and young people with features of BPD to both, research and to the intervention. This feasibility trial, which will recruit from both health services and the community will contribute towards the building evidence base on the complex needs of young people with BPD features who might otherwise be missed from services because they don’t “fit” a traditional service model/diagnosis. This description will be useful to commissioners and policy-makers.

The study has been developed through careful pilot work (29) and collaborations with local service providers, national and international experts in BPD research, academics leading complex clinical trials, youth advisory team, experts by experience and commissioners who are motivated to reduce health inequalities and have a specific focus on mental health resilience for the most vulnerable young people in Scotland. The endeavour to support the feasibility trial in Scotland shows commitment to the mission of early intervention and prevention - not just in principle but through multi-agency collaboration with the endeavour to improve services for some of the most vulnerable children and young people. This feasibility trial includes ongoing consultation with young people with lived experience, and a dissemination plan to bring together commissioners and family members. The outcomes of this feasibility trial will guide a Phase lll clinical trial/definitive RCT.

By conducting a community-based clinical trial with young people, and not limiting the intervention to specialist mental health services, we aim to identify ‘hidden’ young people. This might include, for example, those avoiding contact with services due to previous trauma or neurodevelopmental conditions (for example, ADHD) or complex family needs. We also aim to assess pathways to care and missed opportunities for participation in treatment, and to examine for the possibility of early identification of young people with BPD features in the community, and the possibility of early intervention through joined-up, multi-agency work (schools, youth workers, counsellors, GP’s).

Transparency and data sharing are now widely encouraged for intervention clinical trials, although their application is suboptimal. To promote open science in clinical trials, the fRCT will engage in cross-disciplinary agreement between the Data Monitoring Committee (DMEC) and the Trial Steering Committee (TSC), in consultation with the service user researcher group in NHS Scotland’s Mental Health Network, for a data sharing protocol for a d-RCT. Our syndemics approach is our key methodology for ensuring excellent interdisciplinary working. Syndemics (25) is embedded within the research methodology, the multi-agency intervention approach, our international network of collaborators, and the central role of young people at all stages of study design, delivery and dissemination.

Currently, BPD is viewed erroneously by many clinicians as intractable, with ‘trauma’ as its sole aetiology. Moreover, where specialist services do exist, treatment is usually delayed and/or limited to a select few. Our own pilot study Pathways study’ (29), and work from other groups (58), has shown that, despite the high risk of morbidity and mortality, young people with BPD features are less likely to be assessed and/or treated and often fall into the service gap between child and adolescent and adult mental health services (16). The development of workable, multi-agency services proposed in the trial would be a major advance in optimising care pathways, regardless of trial outcome. Societal and professional attitudes to people living with personality disorders, and to service provision for this vulnerable group, have an important effect upon how services are delivered.

## Limitations

There are systematic, structural barriers to undertaking clinical research with young people living with BPD features. We can therefore never know if we are reaching the entire eligible population. Participants in the trial will not be receiving a diagnosis of BPD. Without a diagnosis, it can be hard for young people to access services, yet, with a diagnosis there is a risk of labelling, stigma and discrimination. We are limited to measure the contribution of structural and individual experiences of stigma and discrimination on engagement and retention within the study. This might mean that eligible participants are excluded by gatekeepers who do not regard them as eligible. We also do not have the resources to actively engage with potentially eligible participants who do not have English as a first language. Although a tool is being developed for the purposes of a treatment fidelity within the f-RCT, there is limited measurement of treatment fidelity for BRIDGE intervention and SAU. No measurement of the content of SAU and no measure of ‘contamination’. The scope of the study limits mapping and modelling of multi-agency service provisions for this group of young people and examining the extent of its contribution to the delivery/completion of the intervention.
